# *Lepr^db/db^* Mice with Senescence Marker Protein-30 Knockout (*Lepr^db/db^Smp30^Y/−^*) Exhibit Increases in Small Dense-LDL and Severe Fatty Liver Despite Being Fed a Standard Diet

**DOI:** 10.1371/journal.pone.0065698

**Published:** 2013-06-03

**Authors:** Yoshitaka Kondo, Goji Hasegawa, Hiroshi Okada, Takafumi Senmaru, Michiaki Fukui, Naoto Nakamura, Morio Sawada, Jo Kitawaki, Takeshi Okanoue, Yuki Kishimoto, Akiko Amano, Naoki Maruyama, Hiroshi Obayashi, Akihito Ishigami

**Affiliations:** 1 Molecular Regulation of Aging, Tokyo Metropolitan Institute of Gerontology, Tokyo, Japan; 2 Department of Endocrinology and Metabolism, Kyoto Prefectural University of Medicine, Graduate School of Medical Science, Kyoto, Japan; 3 Department of Obstetrics and Gynecology, Graduate School of Medical Science, Kyoto Prefectural University of Medicine, Kyoto, Japan; 4 Department of Gastroenterology and Hepatology, Saiseikai Suita Hospital, Osaka, Japan; 5 Institute of Bio-Response Informatics, Kyoto, Japan; Bambino Gesù Children Hospital, Italy

## Abstract

**Background/Aims:**

The senescence marker protein-30 (SMP30) is a 34 kDa protein originally identified in rat liver that shows decreased levels with age. Several functional studies using SMP30 knockout (*Smp30^Y/−^*) mice established that SMP30 functions as an antioxidant and protects against apoptosis. To address the potential role of SMP30 in nonalcoholic fatty liver disease (NAFLD) pathogenesis, we established *Smp30^Y/−^* mice on a *Lepr^db/db^* background (*Lepr^db/db^Smp30^Y/−^* mice).

**Research Design/Principal Findings:**

Male *Lepr^db/db^Smp30^Y/−^* mice were fed a standard diet (340 kcal/100 g, fat 5.6%) for 16 weeks whereupon the lipid/lipoprotein profiles, hepatic expression of genes related to lipid metabolism and endoplasmic reticulum stress markers were analyzed by HPLC, quantitative RT-PCR and western blotting, respectively. Changes in the liver at a histological level were also investigated. The amount of SMP30 mRNA and protein in livers was decreased in *Lepr^db/db^Smp30^Y/+^* mice compared with *Lepr^db/+^Smp30^Y/+^* mice. Compared with *Lepr^db/db^Smp30^Y/+^* mice, 24 week old *Lepr^db/db^Smp30^Y/−^* mice showed: i) increased small dense LDL-cho and decreased HDL-cho levels; ii) fatty liver accompanied by numerous inflammatory cells and increased oxidative stress; iii) decreased mRNA expression of genes involved in fatty acid oxidation (*PPARα*) and lipoprotein uptake (*LDLR* and *VLDLR*) but increased *CD36* levels; and iv) increased endoplasmic reticulum stress.

**Conclusion:**

Our data strongly suggest that SMP30 is closely associated with NAFLD pathogenesis, and might be a possible therapeutic target for NAFLD.

## Introduction

Metabolic syndrome has been described as the association of insulin resistance, hypertension, hyperlipidemia and obesity. Its prevalence has increased dramatically, mainly in developed countries. The hepatic manifestations of metabolic syndrome include nonalcoholic fatty liver disease (NAFLD) and its progressive variant, nonalcoholic steatohepatitis (NASH) [1], [2]. Several animal models have been proposed for NAFLD and NASH research [3]. Since leptin plays a major role in food intake and energy expenditure, total leptin deficiency or leptin resistance can lead to massive obesity, type 2 diabetes, dyslipidemia and fatty liver. Therefore, many investigations pertaining to NAFLD/NASH have been carried out in genetic leptin-deficient ob/ob mice or leptin-resistant db/db mice that were fed a high fat diet (HFD) or the methionine/choline deficiency diet [3]–[5]. However, these models differ significantly from the human NAFLD/NASH phenotype in a number of pathogenically important ways.

The senescence marker protein-30 (SMP30) is a 34 kDa protein that was originally identified in rat liver and its levels decrease with age [6]. We previously reported that SMP30 participates in Ca^2+^ efflux by activating the calmodulin-dependent Ca^2+^-pump that confers resistance to cell injury caused by high intracellular Ca^2+^ concentrations [7]. We identified SMP30 as a gluconolactonase (GNL) that is involved in L-ascorbic acid biosynthesis in mammals, and have established SMP30-knockout (KO) mice [8]. The livers of SMP30-KO mice are highly susceptible to tumor necrosis factor-α (TNF-*a*) and Fas-mediated apoptosis, indicating that SMP30 has an anti-apoptotic effect [9]. SMP30-KO mice showed mitochondrial damage and abnormal accumulation of triglycerides, cholesterol, and phospholipids in the liver [10]. In addition, we reported that decreased SMP30 levels contribute to lowered glucose tolerance [11]. These results are in agreement with several functional studies, which also established that SMP30 functions as an antioxidant and anti-apoptotic protein [12]–[15].

To address the potential role of SMP30 in NAFLD/NASH pathogenesis, we generated SMP30-KO mice on a *Lepr^db/db^* background (*Lepr^db/db^Smp30^Y/−^*) and investigated the lipid/lipoprotein profiles, hepatic expression of genes relevant to lipid metabolism and histological changes in the livers of *Lepr^db/db^Smp30^Y/−^* mice fed a standard diet. Here we show that despite being fed a standard diet, *Lepr^db/db^Smp30^Y/−^* mice have altered lipoprotein components and severe fatty liver accompanied by increased inflammation and oxidative stress induced by mitochondrial and endoplasmic reticulum dysfunction.

## Materials and Methods

### Animal crossing and genotyping, and experimental protocol

**Figure 1:**
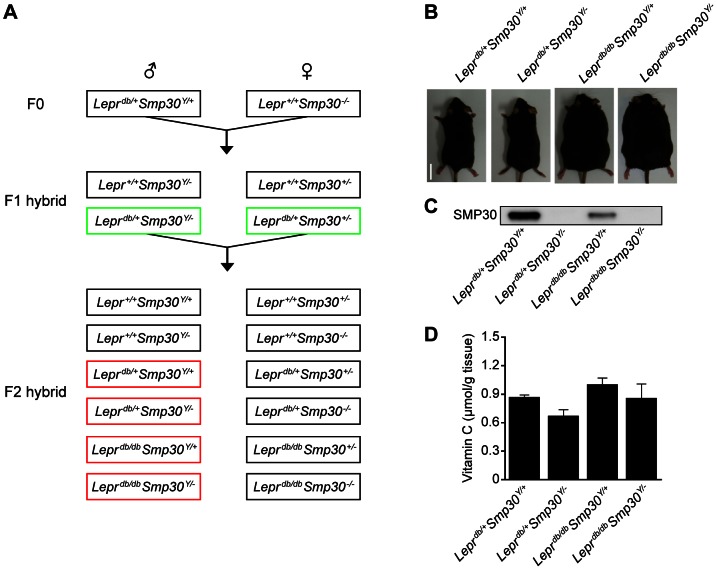
Establishment of *Lepr^db/db^Smp30^Y/−^* mice. (A) Generation of *Lepr^db/db^Smp30^Y/−^* mice. F1 hybrid mice (in green boxes) were crossed to produce the experimental *Lepr^db/+^Smp30^Y/+^*, *Lepr^db/+^Smp30^Y/−^*, *Lepr^db/db^Smp30^Y/+^* and *Lepr^db/db^Smp30^Y/−^* mice (in red boxes). (B) Appearance of *Lepr^db/db^Smp30^Y/−^* mice at 24 weeks of age. (C) Western blot analysis of SMP30 protein levels in livers from each experimental group at 24 weeks of age. (D) Vitamin C concentration in livers from each experimental group of mice at 24 weeks of age. Values are given as means ± SEM of five animals.

We used type 2 diabetic obese *Lepr^db/db^* mice with a C57BLKS/J background. Male *Lepr^db/+^* mice were obtained from Charles River Laboratories Japan, Inc. (Kanagawa, Japan). SMP30-knockout (KO) mice with a C57BL/6 background were established and maintained as described previously [8], [9]. Heterozygous SMP30-KO male mice do not exist, because the *Smp30* gene is located on the X chromosome. SMP30-KO mice cannot synthesize vitamin C *in vivo*, because in mammals SMP30 is the penultimate enzyme in the vitamin C biosynthetic pathway [8]. To maintain vitamin C levels in tissues that were similar to that of wild type mice, and to eliminate any possible confounding influences of vitamin C deficiency, these mice were given free access to water supplemented with 1.5 g/L vitamin C and 10 µM ethylenediaminetetraacetic acid (EDTA) to avoid the effects of vitamin C deficiency [16]. As schematically illustrated in Figure 1A, male *Lepr^db/+^* mice were first crossed with female *Smp30^−/−^* mice to produce male *Lepr^db/+^Smp30^Y/−^* mice and female *Lepr^db/+^Smp30^+/−^* mice. The SMP30 mutant mice genotypes were determined as described previously [9]. Next, male *Lepr^db/+^Smp30^Y/−^* and female *Lepr^db/+^Smp30^+/−^* mice were interbred to produce homozygous *Lepr^db/db^Smp30^Y/+^* and *Lepr^db/db^Smp30^Y/−^* mice and heterozygote control *Lepr^db/+^Smp30^Y/−^* and *Lepr^db/+^Smp30^Y/+^* mice. The mutant *Lepr^db^* gene was identified by restriction enzyme digestion of PCR products. In brief, *Lepr^db^* gene PCR products were amplified by PCR using genomic DNA and forward (5′-AGAACGGACACTCTTTGAAGTCTC-3′) and reverse (5′-CATTCAAACCATAGTTTAGGTTTGTGT-3′) primers. PCR products were then digested by *Afa*I (Takara Bio Inc., Shiga, Japan) and analyzed by agarose gel electrophoresis. The mutant *Lepr^db^* gene showed two bands of 108 bp and 27 bp while the wild type allele showed one 135 bp band.

We prepared four groups of five eight week old male mice, with each group having four genotypes: *Lepr^db/+^Smp30^Y/+^*, *Lepr^db/+^Smp30^Y/−^*, *Lepr^db/db^Smp30^Y/+^*, and *Lepr^db/db^Smp30^Y/−^*. All mice were fed a vitamin C free-standard diet (CL-2; 340 kcal/100 g, fat 5.6%, CLEA Japan, Tokyo, Japan) for 16 weeks. *Lepr^db/+^Smp30^Y/−^* mice and *Lepr^db/db^Smp30^Y/−^* mice had free access to 1.5 g/L vitamin C water containing 10 µM EDTA, whereas *Lepr^db/+^Smp30^Y/+^* and *Lepr^db/db^Smp30^Y/+^* mice had 10 µM EDTA water. Mice were maintained on a 12 h light/dark cycle in a controlled environment. All experimental procedures using laboratory animals were approved by the Animal Care and Use Committee of the Tokyo Metropolitan Institute of Gerontology (Permit Number: 12016).

### Blood and liver tissue collection

All mice were fasted for 16 h and anesthetized at the age of 24 weeks. Blood was obtained from the inferior vena cava, anticoagulated with EDTA, and subsequently centrifuged at 880×*g* for 15 min at 4°C. Mice were systemically perfused with ice-cold phosphate buffered saline through the hepatic portal vein to wash out remaining blood cells and then the livers were removed. The whole body subcutaneous fat was collected and the weight measured. The livers were immersed in RNA*later*® (Life Technologies Corp., Carlsbad, CA, USA) for RNA extraction and fixed with 10% neutral buffered formalin for histological analysis or frozen in liquid nitrogen for biochemical analysis. All samples were stored at −80°C until use.

### Biochemical analysis of blood

Blood glucose levels were measured using a glucometer (Glutest Every; Sanwa Kagaku Kenkyusho Co., Ltd., Aichi, Japan). Plasma insulin levels were measured using an enzyme-linked immunoassay system (Ultra sensitive mouse insulin ELISA kit; Morinaga Institute of Biological Science Inc., Kanagawa, Japan). Plasma total cholesterol (T-cho), triglyceride (TG), phospholipid (PL), aspartate aminotransferase (AST), and alanine aminotransferase (ALT) levels were measured by enzymatic assay kits (Wako Pure Chemicals Industries, Osaka, Japan).

### Western blotting analysis

Livers were homogenized in ice-cold homogenization buffer (10 mM Tris-HCl (pH 8.0), 1 mM EDTA, 1 mM phenyl methanesulfonyl fluoride, and a protease inhibitor cocktail (cOmplete, EDTA-free; Roche Diagnostics GmbH, Mannheim, Germany)) for 30 seconds using a high speed homogenizer (POLYTRON® PT-MR 2100; Kinematica AG, Switzerland). The homogenate was then centrifuged at 21,000×*g* for 10 min at 4°C. The supernatants were boiled for 5 min with a lysis buffer containing 0.125 M Tris–HCl (pH 6.8), 4% SDS, 20% glycerol, 10% 2-mercaptoethanol, and 0.2% bromophenol blue at a ratio of 1∶1. Total protein equivalents for each sample were separated on a 14% polyacrylamide gel and transferred to a polyvinylidene difluoride membrane. The membrane was incubated with the primary antibody, followed by incubation with a horseradish peroxidase-linked goat anti-rabbit IgG (Bio-Rad Laboratories, Tokyo, Japan). The primary antibodies used were: anti-rat SMP30 rabbit polyclonal antibody (Cosmo Bio Co., Ltd., Tokyo, Japan), anti-phosphorylated eukaryotic initiation factor-2α (p-eIF2α) rabbit monoclonal antibody (#3597, Cell Signaling Technology, Beverly, MA) and anti-C/EBP homologous protein (CHOP) rabbit polyclonal antibody (sc-575, Santa Cruz Biotechnology, Santa Cruz, CA) and β-actin (Cell Signaling Technology, Beverly, MA) as a loading control. After washing, immunoreactivity was detected using ECL chemiluminescence reagents (ECL plus, GE Healthcare Japan, Tokyo, Japan). Chemiluminescence signals were quantified with a LAS-3000 imaging system (Fujifilm, Tokyo, Japan). The mean signals from five *Lepr^db/+^Smp30^Y/+^* mice were assigned a relative value of 1.0. The total protein concentration was determined with a Pierce® BCA Protein Assay Kit (Thermo Fisher Scientific Inc., Waltham, MA) using bovine serum albumin as a standard.

### Measurement of vitamin C levels in the liver

Livers were homogenized in 14 vol 5.4% metaphosphate and 1 mM EDTA, and the homogenate was then centrifuged at 21,000×*g* for 15 min at 4°C. Vitamin C was analyzed by high-performance liquid chromatography (HPLC) using an Atlantis dC18 5 µm column (4.6×150 mm; Nihon Waters, Tokyo, Japan) [17]. The mobile phase was 50 mM phosphate buffer (pH 2.8), 0.2 g/L EDTA, and 2% methanol at a flow rate of 1.3 ml/min, and electrical signals were recorded using an electrochemical detector equipped with a glassy carbon electrode at +0.6 V.

### Measurement of T-cho, TG and PL in the liver

Liver tissues were homogenized with 2 vol. of water using a handy homogenizer (Moji-mojikun; Nippon Genetics, Tokyo, Japan). Homogenates were added to a chloroform-methanol (2∶1; v/v) mixture, and centrifuged at 21,000×*g* for 10 min at 4°C. The supernatant organic phase was then collected, dried under nitrogen gas and resolubilized in 2-propanol. T-cho, TG and PL concentrations in total lipid extracts were determined using commercial enzymatic kits (Wako Pure Chemical Industries, Osaka, Japan).

### Thiobarbituric acid reactive substances (TBARS) assay

Lipid peroxidation was estimated by the amounts of TBARS in the liver that were determined according to the method of Ohkawa *et al.* [18]. The livers were first homogenized in ice-cold 0.1 M phosphate buffer (pH 7.4). The homogenates were then centrifuged at 15,000×g for 30 min at 4°C and the supernatant was used for further assays. One volume of sample was mixed thoroughly with two volumes of stock solution (15% (w/v) trichloroacetic acid, 0.375% (w/v) thiobarbituric acid, and 0.25 mM HCl). The mixture was then heated for 30 min in a boiling water bath. After cooling, the flocculate precipitate was removed by centrifugation at 1,000×g for 10 min and the absorbance (OD 532 nm) of the sample was measured. The TBARS levels are expressed as the equivalent amounts of malondialdehyde produced from 1,1,3,3-tetramethoxypropane.

### HPLC analysis of plasma lipoproteins

Plasma lipoproteins were analyzed using a HPLC service (LipoSEARCH) at Skylight Biotech Inc. (Akita, Japan), as previously described [19]. In brief, 10 µL of whole plasma was injected into two connected columns (300×7.8 mm) packed with TSKgel LipopropakXL (Tosoh, Tokyo, Japan) and lipoproteins were separated with 0.05 mol/L Tris-buffered acetate (pH 8.0) containing 0.3 mol/L sodium acetate, 0.05% sodium azide, and 0.005% Brij-35 at a flow rate of 0.7 mL/min. The column effluent was split equally into two lines by a P-460 MicroSplitter (Upchurch Scientific Inc., Oak Harbor, WA, USA); one effluent portion was mixed with cholesterol reagent (Determiner L TC; Kyowa Medex Co., Ltd., Tokyo, Japan) and the other with TG reagent (Determiner L TG; Kyowa Medex). We defined 5 VLDL subclasses (fraction no. 3–7, 30–80 nm), 6 LDL subclasses (fraction no. 8–13, 16–30 nm) and 7 HDL subclasses (fraction no. 14–20, 8–16 nm) using 20 component peaks categorized on the basis of lipoprotein particle size (diameter).

### Histological and immunohistochemical examination of liver

To evaluate histological changes, fixed liver sections were subjected to hematoxylin-eosin (H&E) staining or immunohistochemical staining with an anti-4-hydroxy-2-nonenal (4-HNE) protein adducts monoclonal antibody (1∶40 dilution; Nikken SEIL, Shizuoka, Japan).

The H&E-stained specimens were anonymized, and five different areas per mouse were randomly selected by a researcher. These specimens were scored by two independent investigators blinded to sample identity according to the NASH activity score (NAS) [20] for the degree of steatosis (0–3), lobular inflammation (0–3) and hepatocellular ballooning (0–2). The average of the two investigators' scores was regarded as the score for each mouse.

4-HNE was detected by indirect immunoperoxidase staining using corresponding Histofine Simple Stain MAX-PO kits (Nichirei Biosciences, Tokyo, Japan) and 3, 3-diaminobenzidine (DAB) as a chromogenic substrate. After DAB staining, nuclei were counterstained with Mayer's hematoxylin. Two independent observers evaluated the intensity of 4-HNE immunostaining and assigned scores of 0, 1, 2, or 3 (negative (including faint staining), weak, moderate, or strong, respectively).

### RNA isolation, first-strand cDNA synthesis, and gene expression analysis

Liver tissue was finely ground with a liquid nitrogen-cooled mortar and pestle and homogenized in ice-cold TRIzol reagent (Life Technologies, Carlsbad, CA, USA) before isolation of total RNA according to the manufacturer's instructions. Total RNA (0.5 µg) was reverse-transcribed using PrimeScript RT Master Mix (TaKaRa Bio Inc., Shiga, Japan) for first-strand cDNA synthesis with an oligonucleotide dT primer and random hexamer priming according to the manufacturer's recommendations.

The mRNA expression levels of the following proteins: acetyl-CoA carboxylase (ACC), fatty acid synthase (FAS), sterol regulatory element-binding protein 1c (SREBP1c), SREBP2, 3-hydroxy-3-methylglutaryl-CoA reductase (HMGCoAR), peroxisome proliferator-activated receptor-α (PPARα), medium-chain acyl-CoA dehydrogenase (MCAD), microsomal triglyceride transfer protein (MTP), apolipoprotein-B100 (ApoB100), LDL receptor (LDLR) and VLDL receptor (VLDLR), CD36 and spliced X-box binding protein 1 (sXBP1), all of which are involved in lipid and lipoprotein metabolism, were quantitated using real-time reverse transcription polymerase chain reaction (RT-PCR). RT-PCR was performed using a Thermal Cycler Dice Real Time System II (TaKaRa Bio Inc.) and real-time SYBR® *Premix Ex Taq™* (TaKaRa Bio Inc.) according to the manufacturer's instructions. The specific primers for the target genes and β-actin are described in Table 1. The following PCR conditions were used: 1 cycle for 30 s at 95°C, followed by 40 cycles for 5 s at 95°C, and 30 s at 60°C. The product specificity generated for each primer set was examined for each fragment using a melting curve and gel electrophoresis. The relative expression levels of each targeted gene were normalized to β-actin threshold cycle (CT) values and quantified using the comparative threshold cycle 2^−ΔΔCT^ method as previously described [21]. Signals from *Lepr^db/+^Smp30^Y/+^* mice were assigned a relative value of 1.0. Five mice from each group were examined, and real time RT-PCR was run in duplicate for each sample.

**Table 1:** Primer sequences for use in real-time quantitative RT-PCR.

Gene name		Sequence	Size (bp)	GenBank accession no.
Acetyl-CoA carboxylase	Forward	5′-AGCGACATGAACACCGTACTGAA-3′	106	NM133360
	Reverse	5′-TAGGGTCCCGGCCACATAAC-3′		
Fatty acid synthase	Forward	5′-ATTGGCTCCACCAAATCCAAC-3′	90	NM007988
	Reverse	5′-CCCATGCTCCAGGGATAACAG-3′		
Sterol regulatory element-binding protein-1c	Forward	5′-AGCCTGGCCATCTGTGAGAA-3′	132	NM011480
	Reverse	5′-CAGACTGGTACGGGCCACAA-3′		
Sterol regulatory element-binding protein-2	Forward	5′-CCCACTCAGAACACCAAGCAT-3	67	NM033218
	Reverse	5′-TGGCAGTAGCTCGCTCTCGT-3′		
HMGCoA reductase	Forward	5′- TGTCCTTGATGGCAGCCTTG-3′	183	NM 008255
	Reverse	5′- CCGCGCTTCAGTTCAGTGTC-3′		
Peroxisome proliferator-activated receptor-α	Forward	5′-CTCAGGGTACCACTACGGAGTTCAC-3′	111	NM001113418
	Reverse	5′-TGAATCTTGCAGCTCCGATCAC-3′		
Medium-chain acyl-CoA dehydrogenase	Forward	5′- TGAGTGGCGGCCATTAAGA-3′	179	NM007382
	Reverse	5′- CGGCTTCCACAATGAATCCAG-3′		
Microsomal triglyceride transfer protein	Forward	5′-CATCTCCACAGTGCAGTTCTCACA-3′	167	NM001163457
	Reverse	5′-GGAGTTCACATCCGGCCACTA-3′		
Apolipoprotein-B100	Forward	5′-CAGGTGGCCACAGCCAATAA-3′	161	NM009693
	Reverse	5′-ACTGCAGGTCTGGCTCAGGA-3′		
LDL receptor	Forward	5′- CTAGCAAGTGGGTGTGCGATG-3′	100	NM010700
	Reverse	5′- CTGAATTGATTGGACTGACAGGTGA -3′		
VLDL receptor	Forward	5′- TGGCGTGTGCAAGGCAGTA-3′	145	NM013703
	Reverse	5′- TGCAATGTCCGCATCGAGA-3′		
CD36	Forward	5′-CCCAGATGACGTGGCAAAGA-3′	172	NM001159555
	Reverse	5′-TCCAACAGACAGTGAAGGCTCAA-3′		
Spliced XBP1	Forward	5′-GCTGAGTCCGCAGCAGGT-3′	86	NM013842
	Reverse	5′-GAATCTGAAGAGGCAACAGTGTCA-3′		
Senescence marker protein-30	Forward	5′-GAGGCAGCCTGATGCTGGTAA-3′	92	NM009060
	Reverse	5′-GAGCTGCAGTTCACCCTGCATA-3′		
β-actin	Forward	5′-CATCCGTAAAGACCTCTATGCCAAC-3′	171	NM007393
	Reverse	5′-ATGGAGCCACCGATCCACA-3′		

### Statistical analysis

Data are expressed as means ± SEM. Statistical differences between groups were determined by one-way analysis of variance (ANOVA) with Scheffe's post hoc test. A *P* value<0.05 was considered to be statistically significant.

## Results

### Generation of *Lepr^db/db^Smp30^Y/−^* mice

As shown in Fig. 1A and B, we established *Lepr^db/db^Smp30^Y/−^* mice, which were born at the expected Mendelian ratios and by 24 weeks of age had appearances that were indistinguishable from obese *Lepr^db/db^Smp30^Y/+^* mice. Western blot analysis of liver tissue from *Lepr^db/+^Smp30^Y/−^* and *Lepr^db/db^Smp30^Y/−^* mice demonstrated that these animals lacked SMP30 protein (Fig. 1C). There were no significant differences found in the liver vitamin C concentration among any of the experimental groups (Fig. 1D).

**Figure 2:**
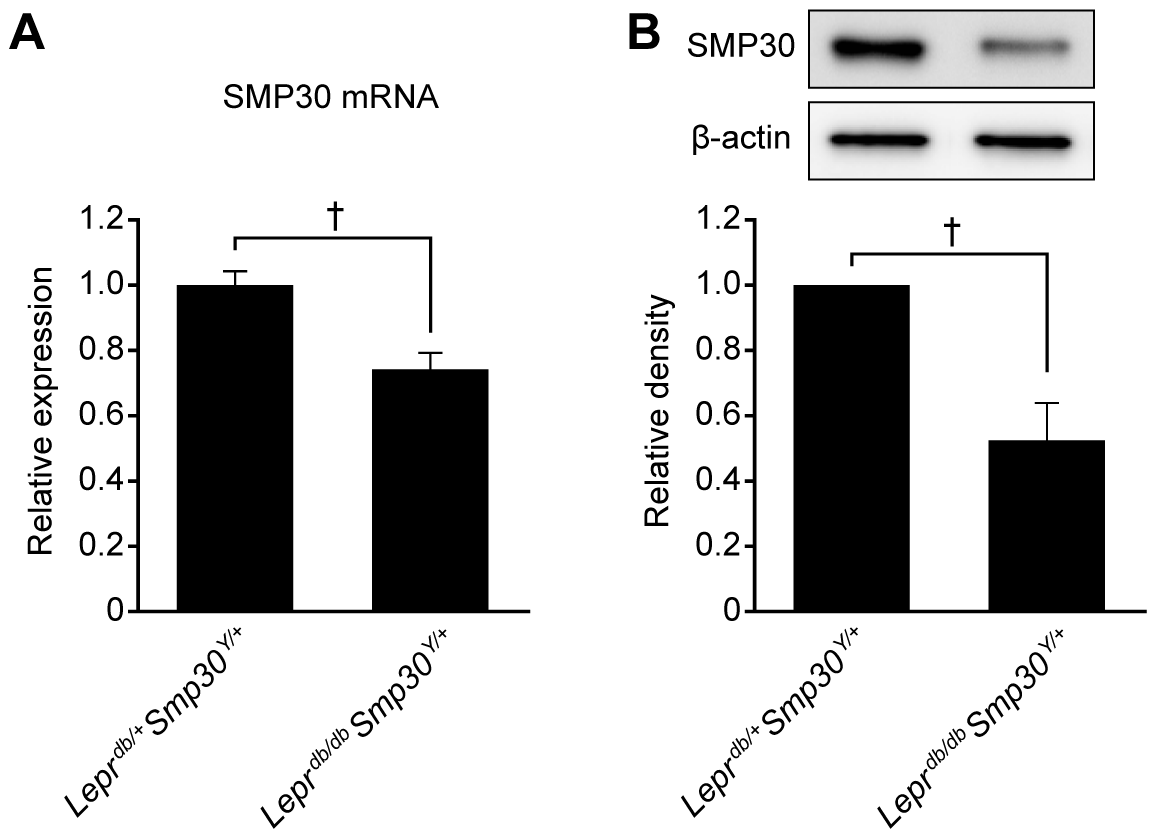
Decrease in hepatic SMP30 levels in *Lepr^db/db^* mice. (A) SMP30 mRNA and (B) protein levels in livers from *Lepr^db/+^Smp30^Y/+^* and *Lepr^db/db^Smp30^Y/+^* mice. Values are given as means ± SEM of five animals. ^†^ *P*<0.01.

Given the difference in SMP30 protein levels observed for *Lepr^db/+^*Smp30^Y/+^ and *Lepr^db/db^Smp30^Y/+^* mice (Fig. 1C), we next quantified the amounts of SMP30 mRNA and protein and found that they were decreased by 25% and 47%, respectively, in *Lepr^db/db^Smp30^Y/+^* mice as compared to *Lepr^db/+^Smp30^Y/+^* mice (both *P*<0.01) (Fig. 2).

### *Lepr^db/db^Smp30^Y/−^* mice had manifestations of metabolic syndrome

The physiological and blood biochemical parameters of 24 week old animals of the four groups are presented in Table 2. Compared with *Lepr^db/+^Smp30^Y/+^* and *Lepr^db/+^Smp30^Y/−^* mice, the food intake of *Lepr^db/db^Smp30^Y/−^* mice during the experimental period was significantly increased by 33% and 40%, respectively (*P*<0.001). Meanwhile, there were no significant differences between *Lepr^db/db^Smp30^Y/−^* and *Lepr^db/db^Smp30^Y/+^* mice (*P* = 0.07). The body weight of *Lepr^db/db^Smp30^Y/−^* mice was 79% and 87% higher than *Lepr^db/+^Smp30^Y/+^* and *Lepr^db/+^Smp30^Y/−^* mice, respectively (*P*<0.001). There were no significant differences in body weight between *Lepr^db/db^Smp30^Y/−^* and *Lepr^db/db^Smp30^Y/+^* mice. Likewise, the epididymal and subcutaneous fat weight in *Lepr^db/db^Smp30^Y/−^* mice were significantly higher than those of *Lepr^db/+^Smp30^Y/+^* and *Lepr^db/+^Smp30^Y/−^* mice (*P*<0.001), although no significant differences in epididymal or subcutaneous fat weight were observed between *Lepr^db/db^Smp30^Y/−^* and *Lepr^db/db^Smp30^Y/+^* mice.

**Table 2:** Physiological, blood and biochemical parameters in four experimetal groups of mice.

	*Lepr^db/+^Smp30^Y/+^*	*Lepr^db/+^Smp30^Y/−^*	*Lepr^db/db^Smp30^Y/+^*	*Lepr^db/db^Smp30^Y/−^*
Food intake (g/day/mouse)	4.2±0.1	3.9±0.1	5.0±0.2§ ^,^ ¶	5.5±0.1§ ^,^ ¶
Body weight (g)	34.4±1.7	32.9±0.9	69.4±2.2§ ^,^ ¶	61.5±3.0§ ^,^ ¶
Epididymal fat weight (g)	1.12±0.18	1.10±0.06	2.60±0.13§ ^,^ ¶	2.42±0.18§ ^,^ ¶
Subcutaneous fat weight (g)	1.20±0.36	1.40±0.09	12.40±0.80§ ^,^ ¶	10.82±0.66§ ^,^ ¶
Fasting blood glucose (mg/dL)	71.4±5.8	82.2±4.3	162.0±15.2* ^,^ #	239.8±26.3§ ^,^ ¶ ^,^ **
Fasting plasma insulin (ng/mL)	1.0±0.2	1.3±0.5	11.6±1.8§ ^,^ ¶	9.2±0.8§ ^,^ ¶
Plasma triglyceride (mg/dL)	33.4±3.7	34.2±3.5	66.6±16.9	78.8±15.6
Plasma cholesterol (mg/dL)	59.6±4.0	58.4±1.7	358.8±58.8§ ^,^ ¶	333.2±68.1§ ^,^ ¶
Plasma phospholipid (mg/dL)	110.8±11.0	125.6±5.2	537.2±44.4§ ^,^ ¶	522.2±53.8§ ^,^ ¶
Plasma aspartate aminotransferase (IU/L)	32.8±4.6	37.6±7.1	98.3±3.7	136.8±27.3† ^,^ ‡
Plasma alanine aminotransferase (IU/L)	11.2±1.5	28.8±14.0	86.3±7.6*	111.0±26.1† ^,^ #

* *P*<0.05,

† *P*<0.01 and

§ *P*<0.001 *versus Lepr^db/+^Smp30^Y/+^*.

# *P*<0.05,

‡ *P*<0.01 and

¶ *P*<0.001 *versus Lepr^db/+^Smp30^Y/−^*.

** *P*<0.05 *versus Lepr^db/db^Smp30^Y/+^*.

Values are given as means ± SEM of five animals.

Fasting blood glucose concentrations were significantly higher in *Lepr^db/db^Smp30^Y/−^* mice (239.8±26.3 mg/dL) than in *Lepr^db/+^Smp30^Y/+^* (71.4±5.8 mg/dL, *P*<0.001), *Lepr^db/+^Smp30^Y/−^* (82.2±4.3 mg/dL, *P*<0.001) and *Lepr^db/db^Smp30^Y/+^* mice (162.0±15.2 mg/dL, *P*<0.05). Fasting plasma insulin concentrations in *Lepr^db/db^Smp30^Y/−^* mice were also higher than in *Lepr^db/+^Smp30^Y/+^* and *Lepr^db/+^Smp30^Y/−^* mice (*P*<0.001). There were no significant differences in fasting plasma levels between *Lepr^db/db^Smp30^Y/−^* and *Lepr^db/db^Smp30^Y/+^* mice.

Although there was a non-significant increase in plasma TG levels in the two groups of *Lepr^db/db^* mice (*Lepr^db/db^Smp30^Y/−^* and *Lepr^db/db^Smp30^Y/+^*) compared with the two groups of *Lepr^db/+^* mice (*Lepr^db/+^Smp30^Y/−^* and *Lepr^db/+^*S*mp30^Y/+^*), there were no significant differences in plasma TG levels among any of the experimental groups. Both plasma T-cho and PL concentrations in *Lepr^db/db^Smp30^Y/−^* mice were significantly higher than those in *Lepr^db/+^Smp30^Y/+^* and *Lepr^db/+^Smp30^Y/−^* mice (both *P*<0.001). There were no significant differences in plasma T-cho and PL concentrations between *Lepr^db/db^Smp30^Y/−^* and *Lepr^db/db^Smp30^Y/+^* mice.

Plasma AST and ALT concentrations in *Lepr^db/db^Smp30^Y/−^* mice (136.8±27.3 and 111.0±26.1 IU/L, respectively) were significantly higher than those of *Lepr^db/+^Smp30^Y/+^* (32.8±4.6 IU/L, *P*<0.01 and 11.2±15 IU/L, *P*<0.01, respectively) and *Lepr^db/+^Smp30^Y/−^* (37.6±7.1 IU/L, *P*<0.01 and 28.8±14.0 IU/L, *P*<0.01, respectively) mice. There were no significant differences in plasma AST and ALT concentrations between *Lepr^db/db^Smp30^Y/−^* and *Lepr^db/db^Smp30^Y/+^* mice.

### *Lepr^db/db^Smp30^Y/−^* mice show decreased HDL-cho and increased small dense LDL-cho levels and increased LDL-cho/HDL-cho ratios

**Figure 3:**
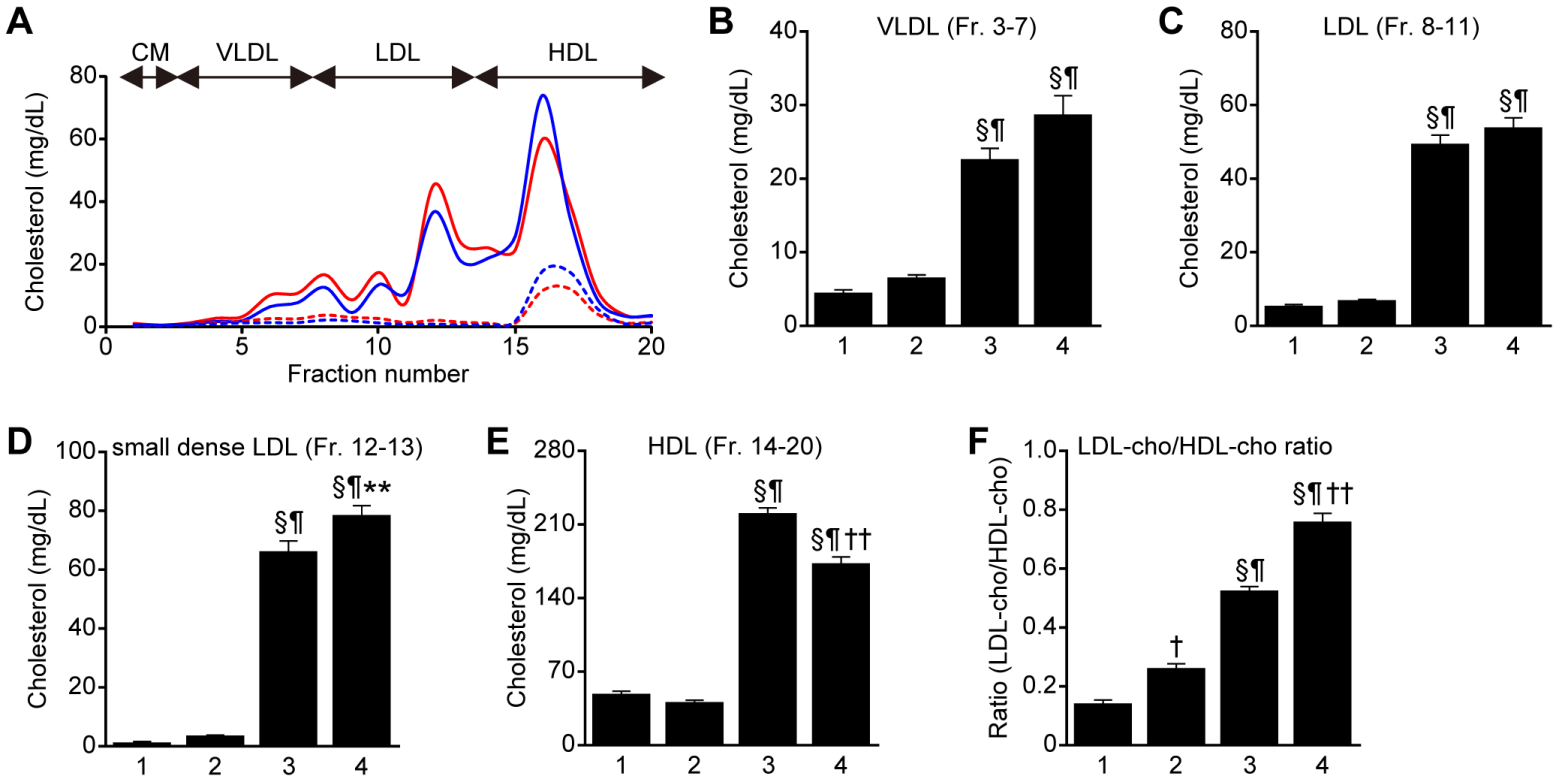
Increase in small dense LDL-cho and decrease in HDL-cho in plasma from *Lepr^db/db^Smp30^Y/−^* mice. (A) HPLC lipoprotein profile with cholesterol reagent and (B–F) cholesterol content in four subfractions according particle size, VLDL; Fr. 3–7, LDL; Fr. 8–11, small dense LDL; Fr. 12–13, HDL; Fr. 14–20 and LDL/HDL ratio in the four experimental groups. Blue dashed line and lane 1, *Lepr^db/+^Smp30^Y/+^* mice; Red dashed line and lane 2, *Lepr^db/+^Smp30^Y/−^* mice; Blue line and lane 3, *Lepr^db/db^Smp30^Y/+^* mice; Red line and lane 4; *Lepr^db/db^Smp30^Y/−^*mice. Values are given as means ± SEM of five animals. ^†^ *P*<0.01 and ^§^ *P*<0.001 *versus Lepr^db/+^Smp30^Y/+^*, ^¶^ *P*<0.001 *versus Lepr^db/+^Smp30^Y/−^*, ^**^ *P*<0.05 and ^††^ *P*<0.001 *versus Lepr^db/db^Smp30^Y/+^*.

HPLC analysis of the plasma lipoprotein profile with cholesterol reagents is shown in Fig. 3A. Either with or without *Smp30*, the levels of VLDL- (Fr. 3–7), LDL- (Fr. 8–11), very-small particle size LDL- (Fr. 12–13) and HDL-cho (Fr.14–20) as well as the LDL-cho/HLD-cho ratio were significantly higher in *Lepr^db/db^* mice (*Lepr^db/db^Smp30^Y/+^* and *Lepr^db/db^Smp30^Y/−^* mice) compared to *Lepr^db/+^* mice (*Lepr^db/+^Smp30^Y/+^* and *Lepr^db/+^Smp30^Y/−^* mice) (*P*<0.001) (Fig. 3B–F). There were no significant differences in VLDL-cho levels in *Lepr^db/db^Smp30^Y/−^* and *Lepr^db/db^Smp30^Y/+^* mice (Fig. 3B). Although there were no significant differences in LDL-cho (Fr. 8–11) levels in *Lepr^db/db^Smp30^Y/−^* and *Lepr^db/db^Smp30^Y/+^* mice (Fig. 3C), the very-small sized LDL-cho particles of Fr.12 and Fr.13 that correspond to small dense LDL-cho (sdLDL-cho) in *Lepr^db/db^Smp30^Y/−^* mice (78.6±3.2 mg/dL) were significantly higher than in *Lepr^db/db^Smp30^Y/+^* mice (66.3±3.5 mg/dL, *P*<0.05) (Fig. 3D), while HDL-cho levels in *Lepr^db/db^Smp30^Y/−^* mice (173.0±6.1 mg/dL) were significantly lower than that of *Lepr^db/db^Smp30^Y/+^* mice (221.0±4.8 mg/dL, *P*<0.001) (Fig. 3E). The LDL-cho/HDL-cho ratio in *Lepr^db/db^Smp30^Y/−^* mice was significantly higher than that in *Lepr^db/db^Smp30^Y/+^* mice (*P*<0.001) (Fig. 3F). Likewise, the LDL-cho/HDL-cho ratio in *Lepr^db/+^Smp30^Y/−^* mice was significantly higher than that of *Lepr^db/+^Smp30^Y/+^* mice (*P*<0.01) (Fig. 3F).

### Increased hepatic lipid (TG, T-cho and PL) and lipid peroxidation (TBARS) levels in *Lepr^db/db^Smp30^Y/−^* mice

**Figure 4:**
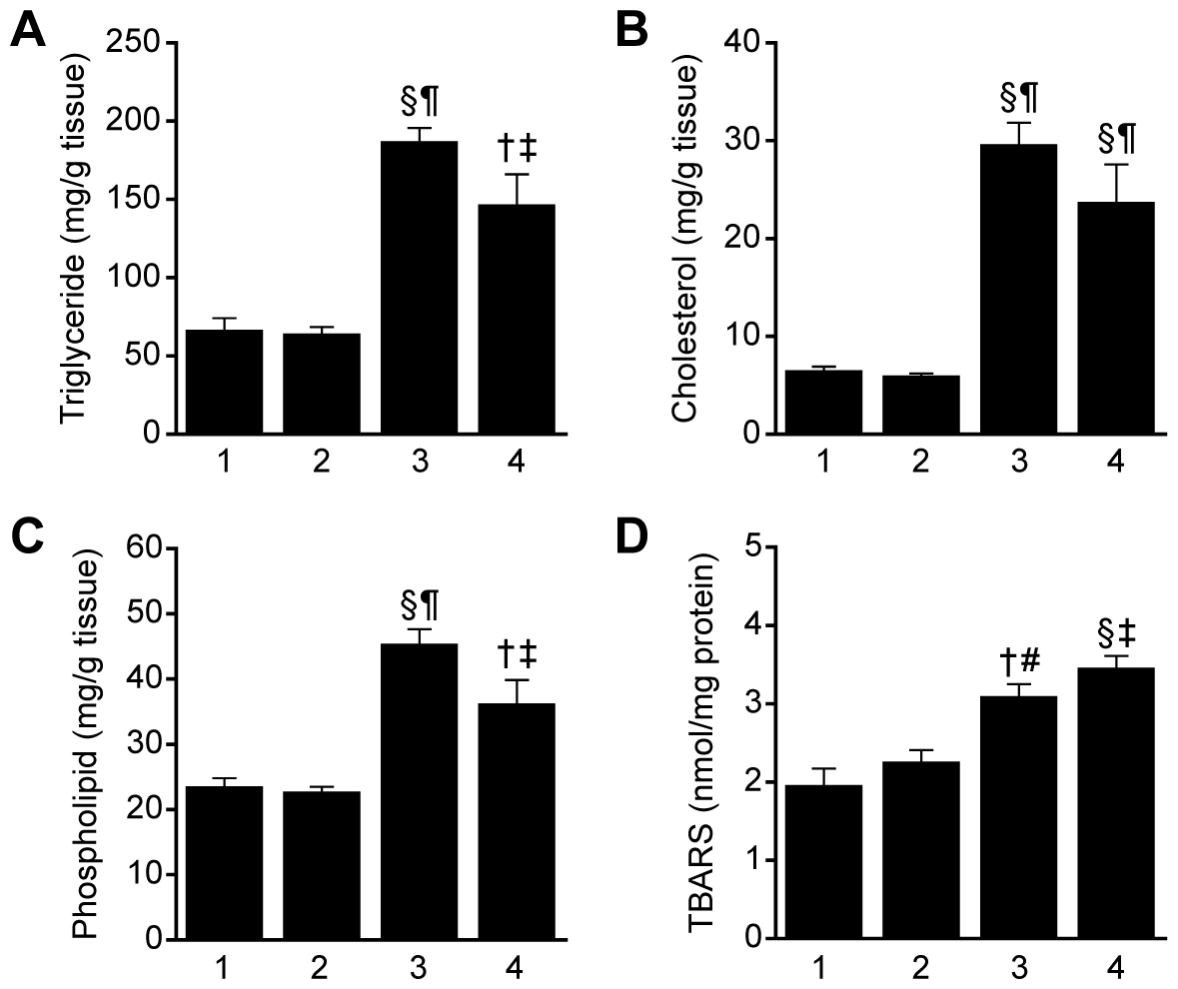
Increase in TBARS in livers from *Lepr^db/db^Smp30^Y/−^* mice. (A) Triglyceride, (B) Cholesterol, (C) Phospholipid and (D) TBARS content in livers from the four experimental groups. Lane 1, *Lepr^db/+^Smp30^Y/+^* mice. Lane 2, *Lepr^db/+^Smp30^Y/−^* mice. Lane 3, *Lepr^db/db^Smp30^Y/+^* mice. Lane 4, *Lepr^db/db^Smp30^Y/−^* mice. ^†^ *P*<0.01 and ^§^ *P*<0.001 *versus Lepr^db/+^Smp30^Y/+^*, ^¶^ *P*<0.001 *versus Lepr^db/+^Smp30^Y/−^*, ^**^ *P*<0.05 and ^††^ *P*<0.001 *versus Lepr^db/db^Smp30^Y/+^*. Values are given as means ± SEM of five animals.

As shown in Fig. 4A–D, hepatic TG, T-cho, PL and TBARS concentrations were significantly higher in *Lepr^db/db^Smp30^Y/−^* mice than *Lepr^db/+^Smp30^Y/+^* or *Lepr^db/+^Smp30^Y/−^* mice (*P*<0.001). Compared with *Lepr^db/db^Smp30^Y/+^* mice, hepatic TG, T-cho and PL concentrations were lower and TBARS concentrations were higher in *Lepr^db/db^Smp30^Y/−^* mice.

### *Lepr^db/db^Smp30^Y/−^* mice show fatty liver accompanied by inflammatory cells and oxidative stress despite being fed a standard diet

**Figure 5:**
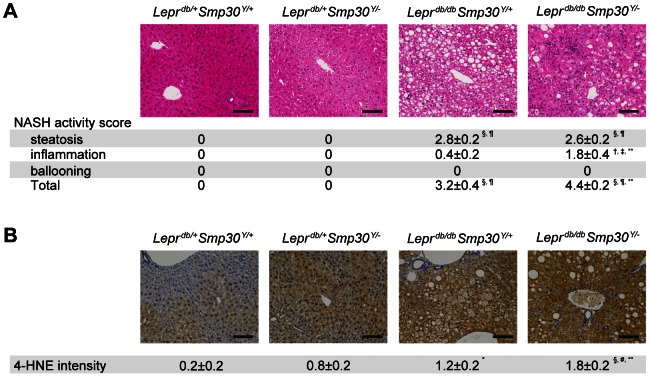
Increased steatosis, inflammation and oxidative stress in liver sections from *Lepr^db/db^Smp30^Y/−^* mice. Representative images of (A) hematoxylin/eosin staining and (B) 4-HNE immunostaining in liver sections from *Lepr^db/+^Smp30^Y/+^*, *Lepr^db/+^Smp30^Y/−^*, *Lepr^db/db^Smp30^Y/+^* and *Lepr^db/db^Smp30^Y/−^* mice. Scale bar is 100 µm. ^*^ *P*<0.05, ^†^ *P*<0.01 and ^§^ *P*<0.001 *versus Lepr^db/+^Smp30^Y/+^*, ^#^ *P*<0.05, ^‡^ *P*<0.01 and ^¶^ *P*<0.001 *versus Lepr^db/+^Smp30^Y/−^*, ^**^ *P*<0.05 *versus Lepr^db/db^Smp30^Y/+^*. Values are given as mean ± SEM of five animals.

As shown in Fig. 5A, hepatic histological examination revealed increased steatosis in *Lepr^db/db^Smp30^Y/+^* mice and increased steatosis accompanied by inflammatory cells in *Lepr^db/db^Smp30^Y/−^* mice. In contrast, no histological abnormalities were observed for both *Lepr^db/+^Smp30^Y/+^* and *Lepr^db/+^Smp30^Y/−^* animals. Fibrosis was not observed in any of the groups.

Regarding the NASH activity score, 24 week old *Lepr^db/db^Smp30^Y/−^* mice had a total score of 4.4±0.2, which was significantly higher than that of *Lepr^db/db^Smp30^Y/+^* mice (3.2±0.4, *P*<0.05). In immunohistochemical examinations using an anti-4HNE antibody, the 4-HNE intensity scores were significantly higher in *Lepr^db/db^Smp30^Y/−^* mice (1.8±0.2) than in *Lepr^db/db^Smp30^Y/+^* (1.2±0.2, *P*<0.05), *Lepr^db/+^Smp30^Y/−^* (0.8±0.2, *P*<0.01) and *Lepr^db/+^Smp30^Y/+^* (0.2±0.2, *P*<0.001) (Fig. 5B) mice.

### Altered hepatic expression of lipid/lipoprotein metabolism-related genes in *Lepr^db/db^Smp30^Y/−^* mice

**Figure 6:**
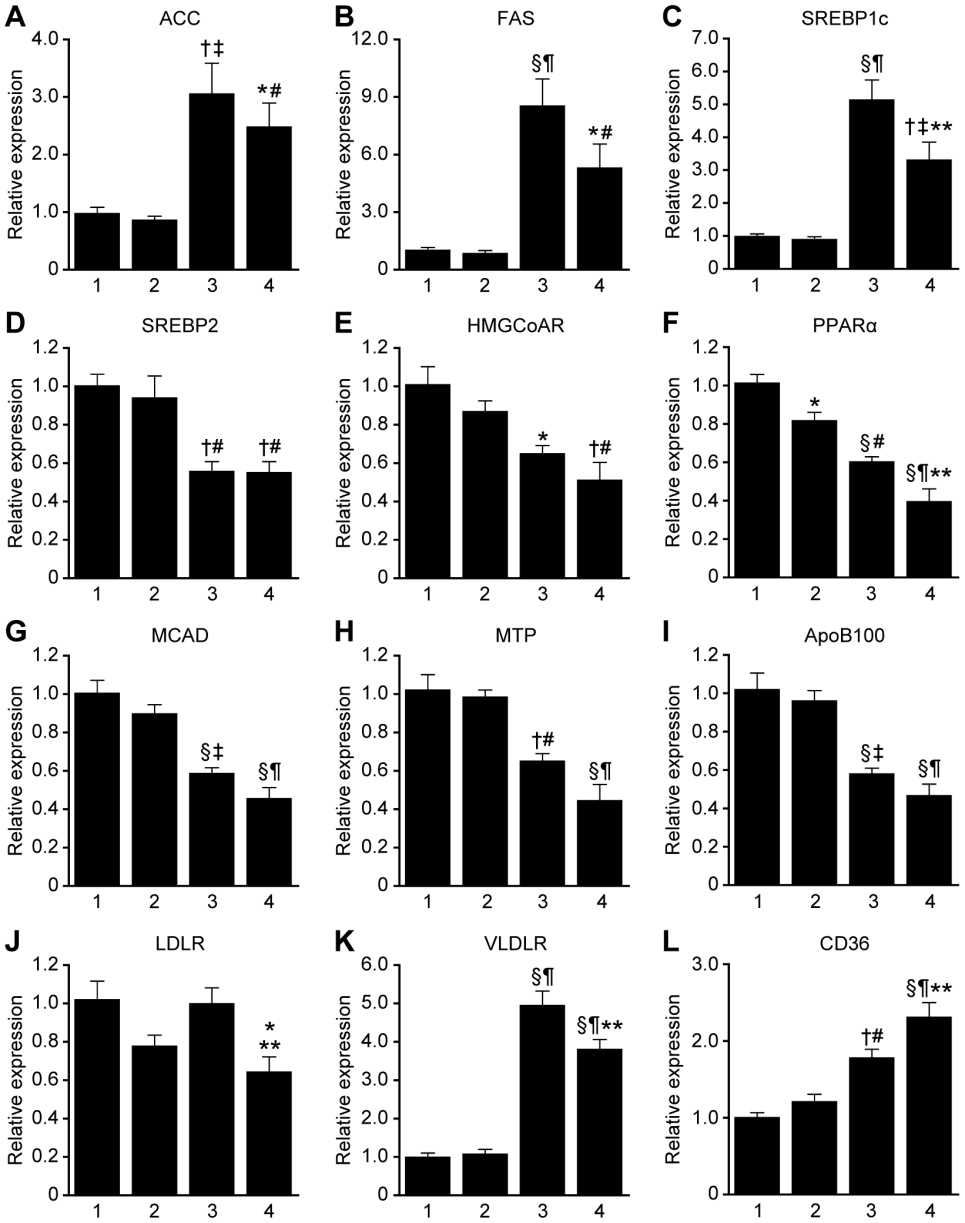
Altered hepatic expression of lipid/lipoprotein metabolism-related genes in *Lepr^db/db^Smp30^Y/−^* mice. Gene expression levels of (A) *ACC*, (B) *FAS*, (C) *SREBP1c*, (D) *SREBP2*, (E) *HMGCoAR*, (F) *PPAR*α, (G) *MCAD*, (H) *MTP*, (I) *ApoB100*, (J) *LDLR* (K) *VLDLR*, and (L) *CD36* in the livers. Lane 1: *Lepr^db/+^Smp30^Y/+^*, lane 2: *Lepr^db/+^Smp30^Y/−^*, lane 3: *Lepr^db/db^Smp30^Y/+^* and lane 4: *Lepr^db/db^Smp30 ^Y/−^* mice. mRNA for each gene was measured using real time RT-PCR and normalized to β-actin. The values from *Lepr^db/+^ Smp30^Y/+^* mice were assigned a relative value of 1.0. Values are given as means ± SEM of five animals. ^*^ *P*<0.05, ^†^ *P*<0.01 and ^§^ *P*<0.001 *versus Lepr^db/+^Smp30^Y/+^*, ^#^ *P*<0.05, ^‡^ *P*<0.01 and ^¶^ *P*<0.001 *versus Lepr^db/+^Smp30^Y/−^*, ^**^ *P*<0.05 *versus Lepr^db/db^Smp30^Y/+^*.

Next, we investigated the hepatic expression of genes related to lipid/lipoprotein metabolism in *Lepr^db/db^Smp30^Y/−^* mice (Fig. 6). Regarding lipogenesis genes, hepatic expression of *ACC*, *FAS* and *SREBP1c* were markedly higher in *Lepr^db/db^* mice (*Lepr^db/db^Smp30^Y/+^* and *Lepr^db/db^Smp30^Y/−^*) than in *Lepr^db/+^* mice (*Lepr^db/+^Smp30^Y/+^* and *Lepr^db/+^Smp30^Y/−^*) with or without the *SMP30* gene. *SREBP1c* mRNA levels in *Lepr^db/db^Smp30^Y/−^* mice were significantly lower than that in *Lepr^db/db^Smp30^Y/+^* mice (*P*<0.05), although there was no significant difference in *ACC* and *FAS* mRNA expression between *Lepr^db/+^Smp30^Y/+^* mice and *Lepr^db/+^Smp30^Y/−^* mice or between *Lepr^db/db^Smp30^Y/+^* mice and *Lepr^db/db^Smp30^Y/−^* mice. On the other hand, independent of the *SMP30* gene, *SREBP2* and *HMGCoAR* mRNA levels were significantly lower in *Lepr^db/db^* mice than those in *Lepr^db/+^*. There were no significant differences in *SREBP2* and *HMGCoAR* expression levels between *Lepr^db/db^Smp30^Y/+^* and *Lepr^db/db^Smp30^Y/−^* mice or between *Lepr^db/+^Smp30^Y/+^* and *Lepr^db/+^Smp30^Y/−^* mice.

mRNA levels of *PPAR*α, which is involved in fatty acid catabolism, were significantly lower in *Lepr^db/db^Smp30^Y/−^* mice than in *Lepr^db/+^Smp30^Y/+^* (*P*<0.001), *Lepr^db/+^Smp30^Y/−^* (*P*<0.001) or *Lepr^db/db^Smp30^Y/+^* (*P*<0.05) mice. Importantly, *PPAR*α mRNA levels were significantly lower in *Lepr^db/+^Smp30^Y/−^* mice compared to *Lepr^db/+^Smp30^Y/+^* mice (*P*<0.05). No significant difference was observed in *MCDA* mRNA levels between *Lepr^db/db^Smp30^Y/+^* and *Lepr^db/db^Smp30^Y/−^* mice or between *Lepr^db/+^Smp30^Y/+^* and *Lepr^db/+^Smp30^Y/−^* mice.

*MTP* and *ApoB100* are key genes for VLDL secretion. Both *MTP* and *ApoB100* mRNA levels were significantly lower in *Lepr^db/db^Smp30^Y/−^* mice than in *Lepr^db/+^Smp30^Y/+^* and *Lepr^db/+^Smp30^Y/−^* mice (*P*<0.001). Compared with *Lepr^db/db^Smp30^Y/+^* mice, *MTP* mRNA expression levels in *Lepr^db/db^Smp30^Y/−^* mice were decreased, although the change did not reach statistical significance. There was no significant difference in *ApoB100* mRNA levels between *Lepr^db/db^Smp30^Y/+^* and *Lepr^db/db^Smp30^Y/−^* mice.

Interestingly, hepatic *LDLR* mRNA levels were significantly lower in *Lepr^db/db^Smp30^Y/−^* mice than in *Lepr^db/+^Smp30^Y/+^* (*P*<0.05) and *Lepr^db/db^Smp30^Y/+^* (*P*<0.05) mice, but not *Lepr^db/+^Smp30^Y/−^* mice. Hepatic VLDLR mRNA levels were significantly higher in *Lepr^db/db^Smp30^Y/−^* mice than in *Lepr^db/+^Smp30^Y/+^* (*P*<0.001) and *Lepr^db/+^Smp30^Y/−^* mice (*P*<0.001), and were significantly lower than in *Lepr^db/db^Smp30^Y/+^* mice (*P*<0.05). *CD36* mRNA levels were significantly higher in *Lepr^db/db^Smp30^Y/−^* mice than in *Lepr^db/+^Smp30^Y/+^* (*P*<0.001), *Lepr^db/+^Smp30^Y/−^* (*P*<0.001) or *Lepr^db/db^Smp30^Y/+^* (*P*<0.05) mice.

### *Lepr^db/db^Smp30^Y/−^* mice show increased endoplasmic reticulum (ER) stress

**Figure 7:**
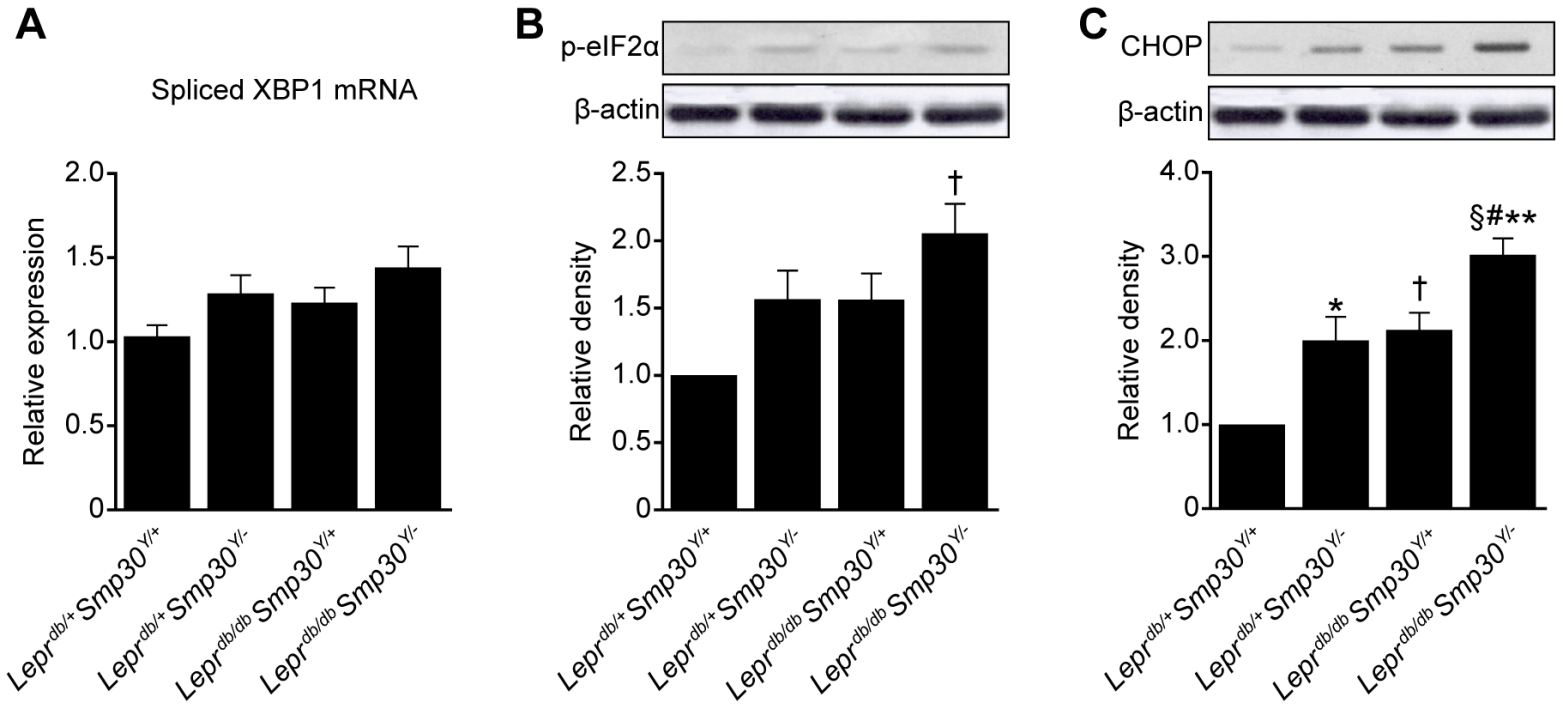
Increase in endoplasmic reticulum (ER) stress in livers from *Lepr^db/db^Smp30^Y/−^* mice. (A) Spliced XBP1 mRNA expression, (B) Phosphorylated eIF2α protein and (C) CHOP protein levels in livers from the four experimental groups. Values are given as means ± SEM of five animals. ^*^ *P*<0.05, ^†^ *P*<0.01 and ^§^ *P*<0.001 *versus Lepr^db/+^Smp30^Y/+^*, ^#^ *P*<0.05 *versus Lepr^db/+^Smp30^Y/−^*, ^**^ *P*<0.05 *versus Lepr^db/db^Smp30^Y/+^*.

The high plasma sdLDL-cho, high liver TBARS content and low mRNA expression levels of fatty acid β oxidation and lipid secretion genes in *Lepr^db/db^Smp30^Y/−^* mice prompted us to analyze hepatic levels of ER stress markers. No significant differences in spliced *XBP1* mRNA expression were observed among the four experimental mice groups (Fig. 7A). Phosphorylated eIF2α (phos-eIF2α) levels in *Lepr^db/db^Smp30^Y/−^* mice were significantly (two-fold) higher (*P*<0.01) compared with *Lepr^db/+^Smp30^Y/+^* mice (Fig. 7B). Also, there was a non-significant increase in phos-eIF2α levels in *Lepr^db/db^Smp30^Y/−^* mice compared with *Lepr^db/+^Smp30^Y/−^* or *Lepr^db/db^Smp30^Y/+^* mice (Fig. 7B). On the other hand, CHOP levels were significantly higher in *Lepr^db/db^Smp30^Y/−^* than in *Lepr^db/+^Smp30^Y/+^* (*P*<0.01), *Lepr^db/+^Smp30^Y/−^* (*P*<0.01) or *Lepr^db/db^Smp30^Y/+^* (*P*<0.05) mice.

## Discussion

The two-hit theory proposed by Day and James [22], in which the initial trigger is the hepatic accumulation of excessive fat, followed by the second hit of oxidative stress, is widely advocated as a pathogenic mechanism for NASH. Therefore, it is of great interest to study in greater detail the role of SMP30 in relation to the pathogenic mechanism for NAFLD/NASH in SMP30-KO mice on a *Lepr^db/db^* background. Interestingly, we observed first that SMP30 levels in *Lepr^db/db^Smp30^Y/+^* mice were significantly lower than in *Lepr^db/+^Smp30^Y/+^* mice, which suggests that this decrease is related to the development of obesity and obesity-related disorders in *Lepr^db/db^Smp30^Y/+^* mice. The mechanism(s) of SMP30 reduction in *Lepr^db/db^Smp30^Y/+^* mouse livers is unknown. Liver SMP30 protein levels were reported to decrease following liver injury in animals treated with carbon tetrachloride [23], lipopolysaccharide (LPS) [24] or D-galactosamine/LPS [25]. Furthermore, we recently reported that 17β-estradiol attenuates saturated fatty acid diet-induced apoptotic liver injury in ovariectomized mice by up-regulating hepatic SMP30 [14]. Thus, the lipid deposition accompanied by increased oxidative and ER stress in *Lepr^db/db^Smp30^Y/−^* mice might result from decreased liver SMP30 levels and in turn exacerbate liver damage via decreased Ca^2+^-pumping activity and anti-oxidative effects of SMP30. Further study will be required to reveal the mechanisms of SMP30 suppression in obesity and obesity-related diseases.

A noteworthy finding of this study is that plasma levels of LDL-cho, in particular the smaller sized particles of Fr.no. 12 and Fr.no. 13 that correspond to small dense LDL-cho (sdLDL-cho) and the LDL-cho/HDL-cho ratio in *Lepr^db/db^Smp30^Y/−^* mice, were significantly higher than those in *Lepr^db/db^Smp30^Y/+^* mice, although plasma levels of T-cho in *Lepr^db/db^Smp30^Y/−^* and *Lepr^db/db^Smp30^Y/+^* mice were similar. A significantly increased LDL-cho/HDL-cho ratio was also found in *Lepr^db/+^Smp30^Y/−^* mice compared with *Lepr^db/+^Smp30^Y/+^* mice. These results indicate that the SMP30 deficiency contributes to increases in plasma sdLDL-cho and decreases in HDL-cho regardless of leptin receptor mutation followed by hyperphagia and obesity. In a human study an association between fatty liver and increased sdLDL-cho was reported [26]–[28]. Furthermore, we recently reported that in patients with histologically diagnosed NAFLD/NASH, serum sdLDL-cho levels in patients with NAS ≥5 were significantly higher than those in patients with NAS≤2, and sdLDL-cho was significantly and inversely correlated with hepatic SMP30 levels [29]. However, we do not presently have an explanation for the observed increase in sdLDL-cho in *Lepr^db/db^Smp30^Y/−^* mice. In humans, TG-rich VLDL (large VLDL1) can be a precursor of sdLDL-cho, i.e., large VLDL1 particles are converted to sdLDL particles by cholesteryl ester transport protein (CETP) and hepatic lipase (HL), the levels of which are commonly increased in type 2 diabetes [30]. Unlike humans, however, mice do not express CETP, and as such, cholesterol is mainly present in HDL. Qiu *et al.* reported that HL-deficient mice have sdLDLs, but TG enrichment was not observed in these mice [31]. In this study, no difference was observed in the mRNA expression of HL between *Lepr^db/db^Smp30^Y/−^* and *Lepr^db/db^Smp30^Y/+^* mice or between *Lepr^db/+^Smp30^Y/−^* and *Lepr^db/+^Smp30^Y/+^* mice (data not shown). Recently, we demonstrated that testosterone-deficient mice fed a high-fat diet showed markedly decreased serum TG and TG-VLDL levels and markedly increased serum sdLDL-cho levels, likely due to altered expression of genes involved in hepatic assembly and lipid secretion [32]. Further work will be required to elucidate the molecular mechanism for this increase in sdLDL-cho in SMP30-KO mice.

An additional notable finding of this study was that although *Lepr^db/db^Smp30^Y/−^* mice showed no advanced stage NASH including fibrosis, compared with *Lepr^db/db^Smp30^Y/+^* mice *Lepr^db/db^Smp30^Y/−^* mice had increased NAS activity and inflammation scores as well as enhanced oxidative stress. Hepatic steatosis results from an imbalance in lipid homeostasis in the liver, where fat uptake, *de novo* lipogenesis, fatty acid oxidation and fat export occur. Compared with *Lepr^db/db^Smp30^Y/+^* mice, RT-PCR of lipid homeostasis-related genes in the liver revealed that the expression levels of these genes, in particular *PPARα* and *SREBP-1c* (but not *CD36*), are reduced in *Lepr^db/db^Smp30^Y/−^* mice. CD36, a member of the class B scavenger receptor family of cell surface proteins, is abundantly expressed in monocytes/macrophages. Therefore, compared with *Lepr^db/db^Smp30^Y/+^* mice, the increase in *CD36* mRNA seen in *Lepr^db/db^Smp30^Y/−^* mice is likely a partial reflection of the increase in the number of inflammatory cells. *PPAR*α mRNA levels were significantly lower not only in *Lepr^db/+^Smp30^Y/−^* mice compared to *Lepr^db/+^Smp30^Y/+^* mice, but also in *Lepr^db/db^Smp30^Y/−^* mice compared to *Lepr^db/db^Smp30^Y/+^* mice, suggesting that decreased hepatic *SMP30* mRNA expression is associated with mitochondrial and peroxisomal fatty acid β-oxidation.

Mitochondrial dysfunction is known to cause increases in oxidative stress, and indeed we showed that the levels of TBARS and 4-HNE rose in the livers of *Lepr^db/db^Smp30^Y/−^* mice. When SMP30-KO mice are fed a vitamin C-deficient diet they do not thrive and display symptoms of scurvy such as bone fractures and rachitic rosary before dying around three months after beginning the deficient diet. As such, *Lepr^db/+^Smp30^Y/−^* and *Lepr^db/db^Smp30^Y/−^* mice were given vitamin C-supplemented water in this study. Vitamin C is known to play an important role in the structure and function of mitochondria and endoplasmic reticulum [33]. Harrison *et al.* reported that vitamin E and vitamin C treatment improves fibrosis but not necroinflammation in NASH patients [34]. Therefore, vitamin C supplementation would be one reason that fibrosis was not observed in *Lepr^db/db^Smp30^Y/−^* mice in this study.

In this study, *Lepr^db/+^Smp30^Y/−^* mice exhibited no phenotypes for lipid accumulation and mitochondrial damage in the liver as was previously reported [10]. However, in the previous study, mice were fed an autoclaved CRF-1 diet containing <55 mg of vitamin C per kg and tap water, and thus the liver vitamin C level in *Smp30^Y/−^* mice was about 6% that of *Smp30^Y/+^* mice [8]. Although lipid accumulation and mitochondrial damage were observed in livers of *Smp30^Y/−^* mice at 12 months [10], it is unclear whether the phenotypes were caused by SMP30 deficiency (loss of unknown function except for vitamin C biosynthesis) and/or by vitamin C deficiency. Thus, the lack of steatosis in *Lepr^db/+^Smp30^Y/−^* mice might result from their younger age (24 weeks) or the vitamin C supplementation in the current study.

Although further studies will be required to define the exact molecular mechanism of the altered lipid homeostasis and liver damage caused by decreases in SMP30 levels, our data strongly suggest that SMP30 is closely associated with NAFLD pathogenesis and might be a possible therapeutic target for NAFLD.

## References

[1] Ong JP, Younossi ZM (2007) Epidemiology and natural history of NAFLD and NASH. Clin Liver Dis 11: 1–16, vii. 17544968 10.1016/j.cld.2007.02.009

[2] Charlton M (2004) Nonalcoholic fatty liver disease: a review of current understanding and future impact. Clin Gastroenterol Hepatol 2: 1048–58. 15625647 10.1016/s1542-3565(04)00440-9

[3] Takahashi Y, Soejima Y, Fukusato T (2012) Animal models of nonalcoholic fatty liver disease/nonalcoholic steatohepatitis. World J Gastroenterol 18: 2300–8. 22654421 10.3748/wjg.v18.i19.2300 PMC3353364

[4] Anstee QM, Goldin RD (2006) Mouse models in non-alcoholic fatty liver disease and steatohepatitis research. Int J Exp Pathol 87: 1–16. 16436109 10.1111/j.0959-9673.2006.00465.x PMC2517349

[5] Rinella ME, Elias MS, Smolak RR, Fu T, Borensztajn J, et al (2008) Mechanisms of hepatic steatosis in mice fed a lipogenic methionine choline-deficient diet. Lipid Res 49: 1068–76. 10.1194/jlr.M800042-JLR200 PMC2311450 18227531

[6] Fujita T, Uchida K, Maruyama N (1992) Purification of senescence marker protein-30 (SMP30) and its androgen-independent decrease with age in the rat liver. Biochim Biophys Acta 1116: 122–128. 1581340 10.1016/0304-4165(92)90108-7

[7] Fujita T, Inoue H, Kitamura T, Sato N, Shimosawa T, et al (1998) Senescence marker protein-30 (SMP30) rescues cell death by enhancing plasma membrane Ca^2+^-pumping activity in HepG2 cells. Biochem Biophys Res Commun 250: 374–380. 9753637 10.1006/bbrc.1998.9327

[8] Kondo Y, Inai Y, Sato Y, Handa S, Kubo S, et al (2006) Senescence marker protein 30 functions as gluconolactonase in L-ascorbic acid biosynthesis, and its knockout mice are prone to scurvy. Proc Natl Acad Sci USA 103: 5723–5728. 16585534 10.1073/pnas.0511225103 PMC1458640

[9] Ishigami A, Fujita T, Handa S, Shirasawa T, Koseki H, et al (2002) Senescence marker protein-30 knockout mouse liver is highly susceptible to tumor necrosis factor-α- and Fas-mediated apoptosis. Am J Pathol 161: 1273–1281. 12368201 10.1016/s0002-9440(10)64404-5 PMC1867294

[10] Ishigami A, Kondo Y, Nanba R, Ohsawa T, Handa S, et al (2004) SMP30 deficiency in mice causes an accumulation of neutral lipids and phospholipids in the liver and shortens the life span. Biochem Biophys Res Commun 315: 575–580. 14975739 10.1016/j.bbrc.2004.01.091

[11] Hasegawa G, Yamasaki M, Kadono M, Tanaka M, Asano M, et al (2010) Senescence marker protein-30/gluconolactonase deletion worsens glucose tolerance through impairment of acute insulin secretion. Endocrinology 151: 529–536. 19934374 10.1210/en.2009-1163

[12] Kondo Y, Sasaki T, Sato Y, Amano A, Aizawa S, et al (2008) Vitamin C depletion increases superoxide generation in brains of SMP30/GNL knockout mice. Biochem Biophys Res Commun 377: 291–296. 18848523 10.1016/j.bbrc.2008.09.132

[13] Handa S, Maruyama N, Ishigami A (2009) Over-expression of Senescence Marker Protein-30 decreases reactive oxygen species in human hepatic carcinoma Hep G2 cells. Biol Pharm Bull 32: 1645–1648. 19801822 10.1248/bpb.32.1645

[14] Fukui M, Senmaru T, Hasegawa G, Yamazaki M, Asano M, et al (2011) 17β-Estradiol attenuates saturated fatty acid diet-induced liver injury in ovariectomized mice by up-regulating hepatic senescence marker protein-30. Biochem Biophys Res Commun 415: 252–257. 22037452 10.1016/j.bbrc.2011.10.025

[15] Sato T, Seyama K, Sato Y, Mori H, Souma S, et al (2006) Senescence marker protein-30 protects mice lungs from oxidative stress, aging, and smoking. Am J Respir Crit Care Med 174: 530–537. 16728709 10.1164/rccm.200511-1816OC

[16] Iwama M, Shimokado K, Maruyama N, Ishigami A (2011) Time course of vitamin C distribution and absorption after oral administration in SMP30/GNL knockout mice. Nutrition 27: 471–478. 20708381 10.1016/j.nut.2010.04.010

[17] Sato Y, Uchiki T, Iwama M, Kishimoto Y, Takahashi R, et al (2010) Determination of dehydroascorbic acid in mouse tissues and plasma by using tris(2-carboxyethyl)phosphine hydrochloride as reductant in metaphosphoric acid/ethylenediaminetetraacetic acid solution. Biol Pharm Bull 33: 364–369. 20190394 10.1248/bpb.33.364

[18] Ohkawa H, Ohishi N, Yagi K (1979) Assay for lipid peroxides in animal tissues by thiobarbituric acid reaction. Anal Biochem 95: 351–358. 36810 10.1016/0003-2697(79)90738-3

[19] Usui S, Hara Y, Hosaki S, Okazaki M (2002) A new on-line dual enzymatic method for simultaneous quantification of cholesterol and triglycerides in lipoproteins by HPLC. J Lipid Res 43: 805–814. 11971952

[20] Kleiner DE, Brunt EM, Van Natta M, Behling C, Contos MJ, et al (2005) Design and validation of a histological scoring system for nonalcoholic fatty liver disease. Hepatology 41: 1313–1321. 15915461 10.1002/hep.20701

[21] Livak KJ, Schmittgen TD (2001) Analysis of relative gene expression data using real-time quantitative PCR and the 2(−Delta Delta C(T)) Method. Methods 25: 402–40. 11846609 10.1006/meth.2001.1262

[22] Day CP, James OF (1998) Steatohepatitis: a tale of two “hits”? Gastroenterology 14: 842–845. 10.1016/s0016-5085(98)70599-2 9547102

[23] Ishigami T, Fujita T, Simbula G, Columbano A, Kikuchi K, et al (2001) Regulatory effects of senescence marker protein 30 on the proliferation of hepatocytes. Pathol Int 51: 491–497. 11472560 10.1046/j.1440-1827.2001.01238.x

[24] Jung KJ, Ishigami A, Maruyama N, Takahashi R, Goto S, et al (2004) Modulation of gene expression of SMP-30 by LPS and calorie restriction during aging process. Exp Gerontol 39: 1169–1177. 15288691 10.1016/j.exger.2004.04.005

[25] Lv S, Wang JH, Liu F, Gao Y, Fei R, et al (2008) Senescence marker protein 30 in acute liver failure: validation of a mass spectrometry proteomics assay. BMC Gastroenterol 8: 17. 18507831 10.1186/1471-230X-8-17 PMC2435529

[26] Toledo FG, Sniderman AD, Kelley DE (2006) Influence of hepatic steatosis (fatty liver) on severity and composition of dyslipidemia in type 2 diabetes. Diabetes Care 29: 1845–1850. 16873790 10.2337/dc06-0455

[27] Cali AM, Zern TL, Taksali SE, de Oliveira AM, Dufour S, et al (2007) Intrahepatic fat accumulation and alterations in lipoprotein composition in obese adolescents: a perfect proatherogenic state. Diabetes Care 30: 3093–3098. 17717283 10.2337/dc07-1088

[28] Sugino I, Kuboki K, Matsumoto T, Murakami E, Nishimura C, et al (2011) Influence of fatty liver on plasma small, dense LDL-cholesterol in subjects with and without metabolic syndrome. J Atheroscler Thromb 18: 1–7. 21041984 10.5551/jat.5447

[29] Park H, Ishigami A, Shima T, Mizuno M, Maruyama N, et al (2010) Hepatic senescence marker protein-30 is involved in the progression of nonalcoholic fatty liver disease. J Gastroenterol 45: 426–434. 19946731 10.1007/s00535-009-0154-3

[30] Adiels M, Borén J, Caslake MJ, Stewart P, Soro A, et al (2005) Overproduction of VLDL1 driven by hyperglycemia is a dominant feature of diabetic dyslipidemia. Arterioscler Thromb Vasc Biol 25: 1697–1703. 15947244 10.1161/01.ATV.0000172689.53992.25

[31] Qiu S, Bergeron N, Kotite L, Krauss RM, Bensadoun A, et al (1998) Metabolism of lipoproteins containing apolipoprotein B in hepatic lipase-deficient mice. J Lipid Res 39: 1661–8. 9717727

[32] Senmaru T, Fukui M, Okada H, Mineoka Y, Yamazaki M, et al (2013) Testosterone deficiency induces markedly decreased serum triglycerides, increased small dense LDL, and hepatic steatosis mediated by dysregulation of lipid assembly and secretion in mice fed a high-fat diet. Metabolism 2013 Jan 16.[Epub ahead of print]. 10.1016/j.metabol.2012.12.007 23332447

[33] Kim JC (1997) Ultrastrutural studies of vascular and muscular changes in ascorbic acid deficient guinea-pigs. Lab Anim 11: 113–117. 10.1258/002367777781005433 68132

[34] Harrison SA, Torgerson S, Hayashi P, Ward J, Schenker S (2003) Vitamin E and vitamin C treatment improves fibrosis in patients with nonalcoholic steatohepatitis. Am J Gastroenterol 98: 2485–2490. 14638353 10.1111/j.1572-0241.2003.08699.x

